# Integrated Genomic Analysis Unveils MicroRNA Roles in Glioma Development

**DOI:** 10.3390/biology15070533

**Published:** 2026-03-27

**Authors:** Sevan Omer Majed, Gaylany H. Abdullah, Kazhal Muhammad Sulaiman, Shawnim M. Maaruf, Raya Kh. Yashooa, Saman S. Abdulla, Chiara Villa, Suhad A. Mustafa

**Affiliations:** 1 Biology Department, College of Education, Salahaddin University-Erbil, Erbil 44001, Kurdistan Region, Iraq; kazhal.sulaiman@su.edu.krd; 2 Medical Research Centre, Hawler Medical University, Erbil 44001, Kurdistan Region, Iraq; gaylany.abdulla@hmu.edu.krd; 3 General Directorate of Scientific Research Center, Salahaddin University-Erbil, Erbil 44001, Kurdistan Region, Iraq; shawnim.maaruf@su.edu.krd; 4 Department of Biology, College of Education for Pure Sciences, University of Al-Hamdaniya, Mosul 41002, Iraq; raya.yashooa@uohamdaniya.edu.iq; 5 College of Dentistry, Hawler Medical University, Erbil 44001, Kurdistan Region, Iraq; samhisto@gmail.com; 6 School of Medicine and Surgery, University of Milano-Bicocca, 20900 Monza, Italy; chiara.villa@unimib.it

**Keywords:** gliomas, non-coding RNAs, expression, microRNAs

## Abstract

Gliomas are the most common type of primary brain tumors in adults, with a high level of recurrence and mortality. Their complex biology and adaptive resistance mechanisms pose major obstacles to existing treatment strategies. Non-coding RNAs (ncRNAs), particularly microRNAs (miRNAs), are crucial in tumor development and progression. Small RNA sequencing technology was performed in 25 patients with high-grade gliomas (HGGs) to analyze ncRNA expression in gliomas compared to normal adjacent tissues (NATs) aiming to elucidate their possible roles in these malignancies. Samples from patients with gliomas were examined, revealing an overall upregulation of ncRNAs. Specific ncRNA classes, including miRNAs, transfer RNAs (tRNAs), Piwi-interacting RNAs (piRNAs), and small nucleolar RNAs (snoRNAs) showed notable shifts in abundance between tumor and normal samples. Among the upregulated miRNAs, a set of top five, such as miR-21, miR-221, miR-1321, miR-1306-5p, and miR-374a-5p, were validated by real-time quantitative PCR (RT-qPCR) in a cohort of 17 low-grade gliomas (LGGs) and 52 HGGs. These miRNAs are associated with critical oncogenic pathways and correlated with a worse prognosis. This study expanded the understanding of glioma biology and further confirmed the role of ncRNAs in the pathogenesis, supporting their potential use as novel possible biomarkers or therapeutic targets. Moreover, it provided an integrated analysis of multiple ncRNA classes, offering validation across both LGG and HGG, and uniquely incorporating a Kurdish cohort.

## Simple Summary

Gliomas are the most common and aggressive among brain cancers. Comparing tumors with normal cerebral tissues, this study explored how the expression of molecules called non-coding RNAs (ncRNAs) contributes to glioma development. The results demonstrated that the levels of these molecules were generally higher in tumors than in normal tissues. Some types of ncRNAs were more common in gliomas, while others were less abundant. These findings are crucial for a better understanding of glioma pathogenesis and may help the identification of potential biomarkers or novel treatment targets.

## 1. Introduction

Gliomas are the most common primary brain neoplasm of the central nervous system (CNS) characterized by a pervasive heterogeneity and hypothesized to originate from glial cells or their precursor cells [1]. They represent 24.8% of all brain and other CNS tumors and account for 82.4% of malignant brain cancers [2]. According to the World Health Organization (WHO) classification system, gliomas are categorized into four grades, based on their histopathological features. Grades I and II are classified as low-grade gliomas (LGGs) while grades III and IV are considered high-grade gliomas (HGGs). LGGs tend to grow slowly and are typically associated with a good overall survival rate. They can originate from any glial cells, with common examples including pilocytic astrocytoma and oligodendroglioma. Conversely, HGGs exhibit rapid proliferation, high infiltrative capacity, and poor prognosis. They include III anaplastic gliomas and grade IV glioblastomas (GBM) [3]. Depending on the tumor’s localization and size, patients may experience a variety of neurological symptoms, including headaches, seizures, sensory deficits, and impairments in speech or vision [4]. Despite the improvement of current available treatments, including surgical resection, radiotherapy, and chemotherapy, the prognosis of patients with gliomas remains unfavorable [5]. Numerous therapeutic challenges, including aggressive growth rates, tumor heterogeneity, and drug resistance, all contribute to its poor prognosis. Therefore, a deeper understanding of the molecular mechanisms underlying gliomas is essential to develop new diagnostic biomarkers and therapeutic approaches.

Although non-coding RNAs (ncRNAs) do not directly participate in protein synthesis, they regulate gene expression at multiple levels, making them potential targets in different cancer types, including gliomas. ncRNAs account for more than 60% of the human genome and are classified according to their structure, activity, biogenesis, position, and crosstalk with DNA or protein-coding mRNAs [6,7]. Based on length, regulatory RNAs are broadly divided into two categories: small ncRNAs, which are less than 200 nucleotides long, and long non-coding RNAs (lncRNAs), which exceed 200 nucleotides. Small ncRNAs (sRNAs) include transfer RNAs (tRNAs), ribosomal RNAs (rRNAs), microRNAs (miRNAs), Piwi-interacting RNAs (piRNAs), small nuclear RNAs (snRNAs), small nucleolar RNAs (snoRNAs), among others [8].

Concerning small ncRNA, miRNAs serve a vital function in regulating gene expression. miRNAs often inhibit gene expression or protein translation by binding to the 3′-UTR of the target mRNAs. miRNAs may cooperate with gene promoters via specific regulators, thereby influencing gene expression through transcriptional regulation [9]. However, interaction of miRNAs with other regions, including the 5′-UTR, gene promoter, and coding sequence, have also been reported, thereby regulating gene expression through multiple mechanisms [10]. They are involved in several physiological and biological processes, such as differentiation, growth, proliferation, apoptosis, and migration [11]. Dysregulated expression of miRNAs is linked to cancer where they may act as oncogenes (oncomiRs) or tumor suppressors by regulating the expression of corresponding genes [12]. OncomiRs downregulate tumor suppressor genes that are normally involved in cell cycle regulation, DNA repair, and apoptosis, resulting in increased expression and uncontrolled cell growth, which promote cancer development and metastasis. In contrast, tumor suppressor miRNAs inhibit oncogene expression, and their loss or downregulation leads to cell proliferation and cancer progression [13]. Several studies have reported the use of miRNAs as biomarkers to diagnose and predict the prognosis of human gliomas, but with some inconsistent and inconclusive results, largely due to different analytical techniques, inappropriate control groups, small sample size and cohort heterogeneity [14,15]. For instance, miR-21 is one of the most frequently upregulated miRNAs in GBM, where it stimulates cell proliferation, invasion, and apoptosis resistance, thus functioning as an oncomiR and correlating with poor prognosis [16,17]. Similarly, miR-10b is involved in increasing tumor invasiveness and is often upregulated HGGs, suggesting its potential role in identifying more aggressive tumor phenotypes [16,18]. By contrast, miR-124, typically enriched in the healthy brain, is markedly downregulated in gliomas, and its restoration inhibits the proliferation and migration of glioma cells, supporting its function as a tumor suppressor [16,19].

This study aims to profile expression of ncRNAs, particularly miRNAs, using small RNA sequencing by comparing tumor samples with matched normal adjacent tissues (NAT), defined as histologically not cancerous tissue located near the tumor site, from glioma patients. The detection of disease-associated ncRNAs could pave the way to identify them as possible biomarkers and therapeutic targets.

## 2. Materials and Methods

### 2.1. Study Design and Patient Cohort

Frozen and formalin-fixed paraffin-embedded (FFPE) tissue specimens were collected from patients with glioma by a surgical assistant at Rizgary Teaching Hospital between October 2024 and February 2025. This study was approved by both Salahaddin University-Erbil (Ref. No. 4sl/39) and Hawler Medical University (Ref. No. 23415 dated on 22 March 2024). All participants were of Kurdish ethnicity and provided written informed consent. Clinical data were collected using a questionnaire, as displayed in Table 1. None of the patients underwent any preoperative chemotherapy or radiotherapy. The overall cohort consisted of 69 patients diagnosed with glioma, including 17 LGGs and 52 HGGs. Matched NATs were obtained approximately 2 cm distant from the tumor margin to ensure the absence of malignant cells and were histopathologically validated.

Due to the high costs, small RNA sequencing was performed as a discovery approach in a subset of 25 HGG patients with matched NAT samples from frozen tissue. This cohort was used to profile global sRNA expression and identify differentially expressed miRNAs. To achieve robust hierarchical clustering analysis, a group of sequencing samples consisting of 12 HGG and 12 matched NAT specimens was chosen based on predetermined quality control criteria, such as data completeness and sequencing quality metrics.

**Figure 1:**
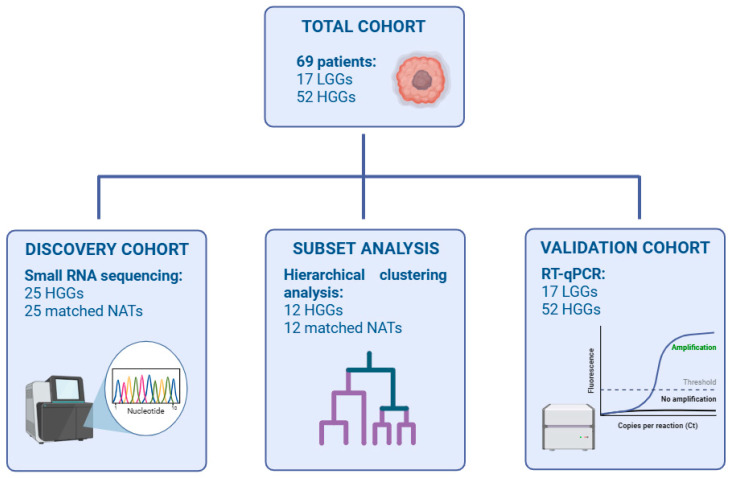
**Study design and patient cohort**. Flow diagram showing patient recruitment, cohort stratification, sequencing discovery set, hierarchical clustering analysis subset, and RT-qPCR validation cohort. Created with BioRender.com.

Five upregulated miRNAs were then validated using real-time quantitative PCR (RT-qPCR) in tumor samples. This validation cohort contained all HGG tumor specimens, incorporating sequencing cases, and 17 independent LGG tumor samples (Figure 1).

### 2.2. ncRNA Expression Analysis by Small RNA Sequencing

Differential expression profiling was performed using TrueQuant technology (GenXPro, Frankfurt, Germany) as a sequencing service. Briefly, total RNA was extracted from frozen tissue sample and processed using the GenXPro small RNA sequencing kit (v1.0), following the manufacturer’s instructions. sRNAs, including miRNAs, siRNAs, snRNAs, snoRNAs, and piRNAs, were then separated using the TrueQuant small RNA kit (GenXPro GmbH, Frankfurt, Germany). Prior to PCR amplification, each sample was labeled using a specific molecular barcode referred to as the unique molecular identifier (UMI) to enable accurate quantification and minimize amplification bias. TrueQuant sRNA libraries were then generated using these barcoded molecules.

For RNA sample library preparation, short RNA transcripts ranging from 15 to 30 bp were isolated from total RNA using the FlashPAGE™ Fractionator System (AM13100) (Life Technologies, Carlsbad, CA, USA) to construct miRNA libraries. The P3 and P5 adapters were specifically ligated to the sRNA transcripts [20]. Single-end sequencing was performed on an Illumina NextSeq500 platform (Illumina, San Diego, CA, USA) with a read length of 1 × 75 bp, achieving a sequencing depth of approximately 30 million reads per sample. A 25-nt UMI-based molecular barcoding strategy was used to label the sRNA transcripts. Raw reads were processed to remove low-quality sequences and flanking adapter regions. The filtered reads were then aligned to the reference genome using the Bowtie 2 tool aligner [21], and subsequently annotated with relevant transcript features. The quantification of gene-level read counts was performed using HTSeq, followed by differential expression analysis with DESeq2, which applies negative binomial generalized linear models [22]. A final table of results was generated, including key statistical metrics such as *p*-values, false discovery rate (FDR), and log2 fold changes (log2FC). Mature miRNAs were annotated using miRBase. For visualization purposes, expression levels of sRNA classes were normalized as reads per million (RPM).

### 2.3. Bioinformatic Analysis

Quality control of raw sequencing reads was performed using FastQC (version 0.12.0) to assess per-base sequence quality, GC content, and potential adapter contamination. Low-quality bases indicated with a Phred score below 20, were trimmed, and reads shorter than 50 bp after trimming were discarded. Adapter sequences were also removed prior to downstream analyses. The resulting high-quality and cleaned reads were then aligned to the GRCh38/hg38 reference genome using the Bowtie 2 tool (version 2.5.5), allowing a maximum of two mismatches per read during alignment [23], which balances sensitivity for detecting sRNAs and specificity to minimize false positives. Differential expression analysis was performed with DESeq2 package (version 1.50.1) in R, which applies a negative binomial generalized linear model and the Wald test to estimate differential expression [24]. The Benjamini–Hochberg procedure in the multitest package was used to adjust *p*-values and control the FDR. Differentially expressed sRNAs were identified using cutoff criteria of FDR < 0.05 and |log2FC| > 0.5. Volcano plots were generated based on log2FC and adjusted *p*-values.

To identify potential candidate target genes possessing binding site to 3′-UTR of the miR-21, miR-221, miR-1306, miR-1321, and miR-374a, five predicted databases were used: MirBase, miRTarBase, miRWalk, Target Scan, and MirTar2. The prognostic relevance of candidate miRNAs was evaluated using Kaplan–Meier Plotter (https://kmplot.com/analysis/; last access on 3 September 2025) and GEPIA2 (http://gepia2.cancer-pku.cn/#index, accessed on 3 September 2025). Overall survival analysis was performed using data from 250 glioma patients included in The Cancer Genome Atlas (TCGA) dataset, based on Gene Chip miRNA expression data. Survival curves were generated using the Kaplan–Meier method, and statistical significance was assessed using the log-rank test.

### 2.4. miRNA Validation by RT-qPCR

To validate the TrueQuant sequencing results, the expression levels of the top five significant upregulated miRNAs, namely miR-21-5p, miR-221-5p, miR-1321, miR-1306-5p, and miR-374a-5p, were quantified via RT-qPCR technique. Total RNA was extracted by FFPE tissue specimens, using the FFPE RNA/DNA Purification Plus kit (Norgen Biotek Corp., Thorold, ON, Canada), according to the manufacturer’s instructions. RNA concentration and purity were evaluated with a Nanodrop ND2000 spectrophotometer (Thermo Fisher Scientific, Waltham, MA, USA).

Complementary DNA (cDNA) was synthesized using miRNA All-In-One cDNA Synthesis Kit (Abmgood, Richmond, BC, Canada). RT-qPCR was performed using BrightGreen miRNA qPCR MasterMix-ROX (Cat. No. MasterMix-mR) on a CFX96 Real-Time PCR Detection System (BioRad, Puchheim, Germany). Commercially available primers for miR-21-5p (MPH02337), miR-221-5p (MPH02351), miR-1321 (MPH01273), miR-1306-5p (MPH02167), and miR-374a-5p (MPH02558) were purchased from Abmgood (Richmond, BC, Canada). SNORD44 (MPH0005) and U6-2 (MPH0001) (Abmgood, Richmond, BC, Canada) were used as endogenous controls. The reaction was performed using the following 3-step cycling program: initial enzyme activation at 95 °C for 10 min, followed by 35 cycles of denaturation at 95 °C for 10 s, annealing at 60 °C for 20 s, and extension at 72 °C for 20 s. Relative quantification of miRNA expression levels was determined using the comparative Ct (2^−ΔΔCt^) method, with normalization to SNORD44 and U6-2. All samples were run in duplicates and experiments were repeated independently three times. The stability of the reference miRNAs was assessed using geNorm algorithm, which confirmed their suitability as housekeeping controls.

### 2.5. Statistical Analysis

Statistical analyses were carried out using R (version 3.6.1) and GraphPad Prism (version 9.4.1). For RT-qPCR validation experiments, differences between groups were assessed using a two-tailed unpaired Student’s *t*-test. A *p*-value < 0.05 was considered statistically significant. Data are expressed as the mean ± standard deviation (SD).

Pearson’s distance metric and average linkage were used for hierarchical clustering analysis of miRNA expression data using GENE-E software (version 2.12) (available at https://software.broadinstitute.org/GENE-E/index.html, accessed on 2 January 2026). Principal component analysis (PCA) was performed in R using the prcomp function on log2-transformed and z-score-normalized expression data to evaluate global variance and group-specific expression pattern [25].

## 3. Results

### 3.1. Comprehensive Analysis of Genomic Regions

**Figure 2:**
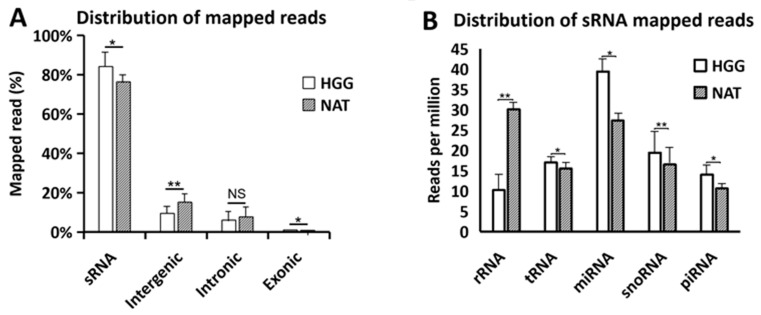
**Distribution of annotated transcripts in HGG against NAT.** (**A**) Proportion of mapped reads categorized as sRNA, intergenic, intronic, and exonic transcripts. (**B**) Relative distribution of sRNA classes expressed as reads per million (RPM). Statistical significance is denoted as follows: NS for not significant, * for *p* ≤ 0.05, and ** for *p* ≤ 0.01.

Overall, the distribution of total mapped reads between HGG and NAT samples is shown in Figure 2. They were categorized into sRNA, intergenic, intronic, and exonic transcripts (Figure 2A). Compared to the total mapped reads, sRNA molecules represented the predominant fraction in both groups, accounting for 82.4% in HGG and 79.0% in NAT. In contrast, intergenic transcripts amounted for 8.6% in HGG and 10.0% in NAT, while intronic reads represented 7.0% and 8.0%, respectively. Exonic reads constituted only a minor fraction in both groups.

The relative distribution of various classes of sRNA molecules is shown in Figure 2B. In HGG samples, the miRNA family was the most prevalent sRNA class (39.4 reads per million, RPM), followed by snoRNAs (19.4 RPM) and tRNAs (17.0 RPM), while piRNAs (14.0 RPM) and rRNAs (10.2 RPM) were less represented. In contrast, NAT samples exhibited a markedly higher abundance of rRNAs (30.1 RPM), making it the most abundant sRNA class in this group. MiRNAs were the second most represented class (27.3 RPM), followed by snoRNAs (17.0 RPM), tRNAs (15.0 RPM), and piRNAs (8.5 RPM).

### 3.2. Comprehensive Analysis of ncRNAs

**Figure 3:**
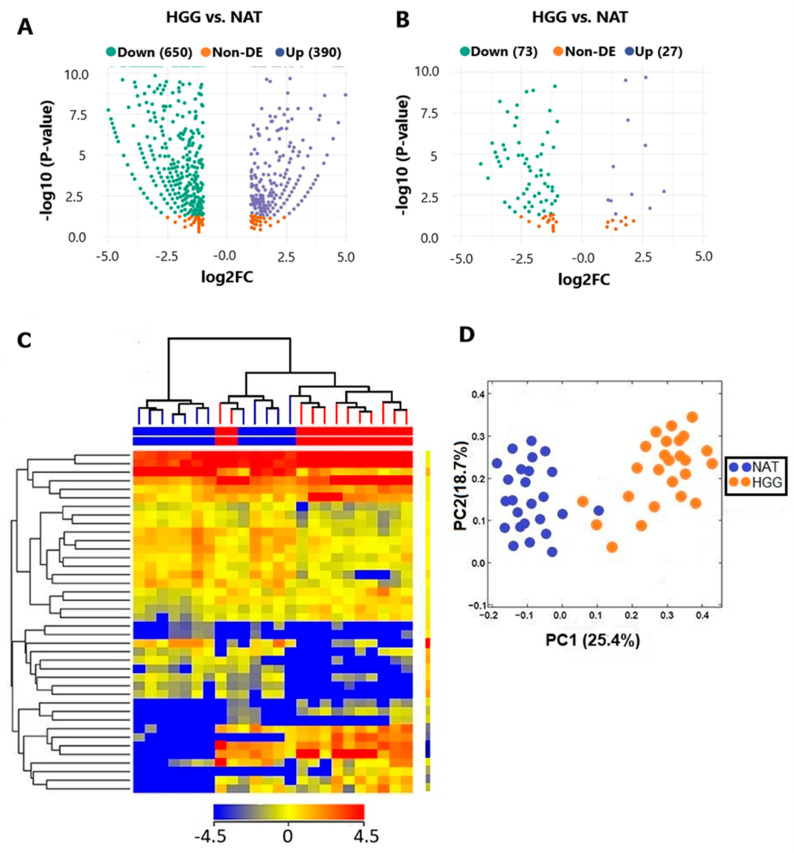
**Differentially expressed sRNA analyses.** (**A**) Differential expression of total sRNA molecules in HGG was displayed, as compared to NAT. (**B**) Differentially expressed and the most significantly overexpressed miRNAs were shown in HGG against NAT, with a *p*-value less than 0.05. (**C**) Hierarchical clustering of differentially expressed miRNAs showing the separation between HGG and NAT samples. Rows represent miRNAs and columns represent individual samples. The color scale indicates relative miRNA expression levels. (**D**) PCA of miRNA expression profiles illustrating the separation between HGG and NAT samples.

Differentially expressed levels of annotated sRNAs between HGG and NAT are shown in Figure 3A. A total of 650 were found to be downregulated, while 390 were downregulated. In addition, over 200 sRNA exhibited non-differential expression (non-DE). Specifically, among the sRNA molecules, 73 miRNAs were downregulated and 27 were upregulated in HGG compared with NAT (Figure 3B, Table 2). Hierarchical clustering analysis based on differentially expressed miRNAs was performed on 12 HGG and 12 matched NAT samples. Results revealed a clear segregation of samples into two distinct clusters, reflecting markedly different miRNA expression profiles between HGG and NATs (Figure 3C). To further investigate global miRNA expression patterns, PCA was performed (Figure 3D), demonstrating a clear separation between the two groups according to pathological status. The two principal components accounted for 44.1% of the total variance (PC1: 25.4%; PC2: 18.7%), indicating that variations between HGG and NAT samples are responsible for a significant amount of the dataset’s variability (Figure 3D).

### 3.3. Validation of Top Five Upregulated miRNAs by RT-qPCR

**Figure 4:**
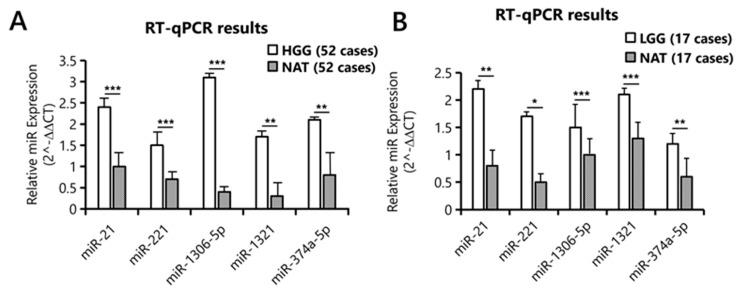
**Validation of differentially expressed miRNAs.** Relative quantification of miRNA expression in HGG (**A**) and LGG (**B**) versus NAT by RT-qPCR. Statistical significance is denoted as follows: * for *p* ≤ 0.05, ** for *p* ≤ 0.01, and *** for *p* ≤ 0.001.

Among all sRNA classes, miRNAs were selected for validation, due to their role as key regulators of gene expression. Specifically, only upregulated miRNAs were exclusively chosen, given their greater potential for targeted inhibition or pharmacological modulation. The significant upregulation of selected candidates was confirmed in all cases of the HGG cohort: miR-21 (*p* = 0.001), miR-221 (*p* = 0.004), miR-1306-5p (*p* = 0.002), miR-1321 (*p* = 0.03), and miR-374a-5p (*p* = 0.01) (Figure 4A). To investigate whether their dysregulation may be involved early in gliomagenesis, the expression of these miRNAs was further analyzed in 17 LGG samples. Data confirmed their significant upregulation in this cohort as well: miR-21 (*p* = 0.02), miR-221 (*p* = 0.05), miR-1306-5p (*p* = 0.001), miR-1321 (*p* = 0.007), and miR-374a-5p (*p*= 0.01) (Figure 4B).

### 3.4. Prediction of Candidate Target Genes Regulated by the Selected miRNAs

To elucidate the potential molecular interactions of the selected miRNAs, candidate target genes were predicted by integrating results from five independent computational databases (miRBase, TargetScan, miRTar, miRMap, and miRDB). For each miRNA, three high-confidence putative target genes harboring complementary binding sites within the 3′-UTR were prioritized. Details of these predicted targets are summarized in Table 3.

### 3.5. Prognostic Significance of Candidate miRNAs in Glioma

**Figure 5:**
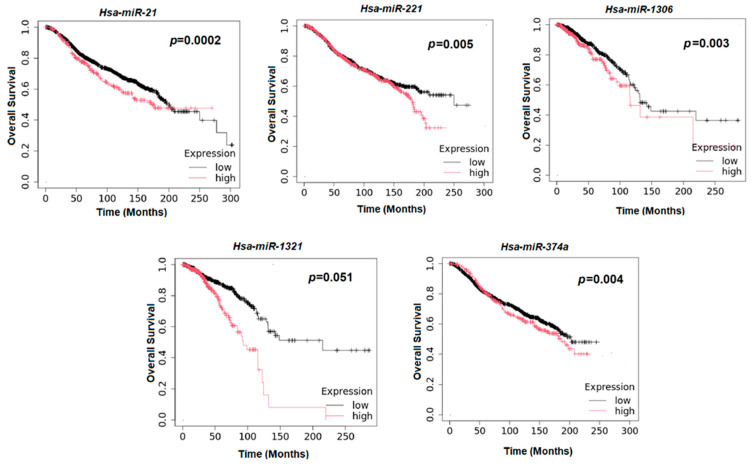
**Kaplan–Meier overall survival analysis of glioma patients from the TCGA cohort.** Patients (n = 250) were stratified into high- and low-expression groups. Elevated miR-21, miR-221, miR-1306, and miR-374a-5p levels were associated with shorter overall survival, whereas miR-1321 showed a non-significant trend (log-rank test).

Kaplan–Meier survival analyses were performed on 250 glioma patients from the TCGA dataset to assess the prognostic value of potential miRNAs. Patients were stratified into high- and low-expression groups based on their miRNA expression levels. High miR-21 expression was significantly associated with reduced overall survival (*p* = 0.0002). Similarly, elevated levels of miR-221 (*p* = 0.005), miR-1306 (*p* = 0.003), and miR-374a-5p (*p* = 0.004) were significantly associated with poorer survival outcomes. In contrast, although patients with high miR-1321 expression showed a trend towards decreased overall survival, this association did not reach statistical significance (*p* = 0.051), Overall, these findings indicate that increased expression of specific miRNAs is associated with unfavorable prognoses in glioma patients (Figure 5).

## 4. Discussion

NcRNAs are increasingly recognized as critical regulators of cellular processes, particularly in cancer development and progression [26]. Using integrated analysis, this study demonstrated that ncRNAs are globally dysregulated in gliomas compared to normal tissues. The use of matched NAT samples minimizes inter-individual variability and increases the biological significance of the observed expression variations, implying that the discovered changes in ncRNAs are tumor-specific rather than patient-dependent. Accordingly, unsupervised analyses clearly revealed that NAT and HGG samples segregate into two distinct molecular clusters. The hierarchical clustering demonstrated a consistent and robust separation between the two groups, implying that the chosen expression profile reflects underlying biological differences rather than random variability. Importantly, the PCA revealed a concordant separation, indicating that the pathological status of the samples is the primary source of variability in our dataset.

Among the differentially expressed molecules, miRNAs emerged as the most prominently dysregulated class. Specifically, this study identified five significantly upregulated miRNAs, such as miR-21, miR-221, miR-1306-5p, miR-1321, and miR-374a-5p, which were validated by RT-qPCR, confirming the reliability of the sequencing results. Notably, their overexpression was confirmed in both LGG and HGG samples, suggesting that their dysregulation may be implicated early in glioma development rather than being strictly associated with tumor grade and aggressiveness. The cross-grade validation supports the broader biological significance of the identified upregulated miRNAs, even if direct global comparisons across tumor grades were not feasible in the sequencing analysis. Importantly, Kaplan–Meier survival analysis using TCGA datasets demonstrated that increased expression of miR-21, miR-221, miR-1306-5p, and miR-374a-5p was strongly associated with poor overall survival, suggesting their clinical relevance as prognostic biomarkers. Although miR-1321 did not reach statistical significance, the observed trend suggests a possible biological implication requiring further investigation in larger cohorts. While miR-21 and miR-221 are well-established oncomiRs involved in glioma invasion, migration, angiogenesis, and treatment resistance [27,28,29,30], our findings are consistent with previous studies and provide independent validation of their prognostic value within our cohort, emphasizing their importance in glioma biology. Elevated plasma levels of miR-221 have also been proposed as predictive biomarkers, highlighting its potential utility in both diagnosis and prognosis [31]. Concerning miR-374a, it has been shown that its knockdown enhances etoposide-induced cytotoxicity against glioma cells through overexpression of *FOXO1*, a reported tumor suppressor in multiple cancers [32]. Conversely, the involvement of miR-1306-5p and miR-1321 in glioma pathogenesis remains less well-characterized. Although little is known about miR-1306-5p in gliomas, studies in other tumor types have linked this miRNA to pathways regulating proliferation and apoptosis [33]. Similarly, miR-1321, has been reported to be upregulated in pediatric gliomas [34]. Taken together, their significant upregulation in our cohorts, combined with their prognostic associations, suggested novel roles in gliomas that require further functional investigation. In silico prediction of miRNA–mRNA interactions identified several putative target genes of biological relevance, further strengthening the potential functional importance of upregulated miRNAs. For instance, miR-21 was predicted to regulate *ZNF367*, *NFAT5*, and *PIK3R1*, linking it to PI3K signaling and cell cycle control [35,36]. MiR-221 was predicted to target *TP53I11*, which suppressed epithelial–mesenchymal transition and metastasis in breast cancer cells [37]. These bioinformatic findings suggest that the identified miRNAs may function as upstream modulators of gene regulatory pathways relevant to glioma pathogenesis, such as tumor growth, cell cycle regulation, survival signaling, and cellular adaptation to stress. While these predictions require experimental validation, they provide a useful framework for future studies.

Conversely, several miRNAs, including miR-1, miR-29a, miR-128, and miR-139-5p, which have previously been linked to tumor suppression, were found to be significantly downregulated. Their decreased expression in our cohort is consistent with a loss of inhibitory control over proliferation, stemness, and therapy resistance pathways [38,39,40,41,42,43]. Our dataset shows that oncogenic miRNAs are upregulated while tumor suppressor miRNAs are downregulated, indicating a coordinated changing of post-transcriptional regulatory networks that may collectively drive glioma growth.

Beyond miRNAs, the sequencing analysis also revealed widespread dysregulation of other ncRNAs, including rRNAs, tRNAs, snoRNAs, and piRNAs. Although not functionally validated in the present study, these findings further highlight the complexity of gene regulation in glioma. Their abnormal expression is involved in the occurrence and development of tumors through different mechanisms, such as transcriptional inhibition and post-transcriptional regulation [44]. For example, snRNAs primarily guide post-transcriptional modifications of rRNAs and tRNAs, influencing their structure and function, thereby affecting cellular homeostasis [45]. Similar to miRNAs, piRNAs have both oncogenic and tumor suppressive roles in cancer development [46].

The potential of ncRNAs as biomarkers for glioma diagnosis and therapeutic targets is particularly promising. For instance, strategies aiming to inhibit oncomiR or restore tumor suppressor miRNAs could provide new avenues for targeted therapy [27,47,48,49]. Despite these insights, this study has several limitations. Firstly, due to resource constraints, sequencing analysis was limited to HGG samples, preventing global expression comparisons across tumor grades. The use of samples from a single ethnic group within one clinical center may restrict the generalizability of our findings. Additionally, while this study focuses on the differential expression of ncRNAs, the functional roles of many identified ncRNAs remain to be elucidated. Future research should include functional validation of key ncRNAs using in vitro and in vivo models and explore the mechanisms by which these ncRNAs regulate glioma progression [50,51]. The lack of molecular characterization, including IDH mutation status, 1p/19q codeletion, ATRX, and TP53 alterations, which the 2021 WHO CNS classification requires for accurate diagnosis and prognostic stratification of gliomas, is a significant limitation of the study. The results are based solely on histological criteria and should therefore be interpreted in this context.

Overall, this study provides a comprehensive overview of ncRNA dysregulation in gliomas, highlighting their dual roles as oncogenic and tumor suppressor factors. These findings pave the way for future research to develop ncRNA-based diagnostics and therapeutics, potentially leading to improved glioma management strategies [52].

## 5. Conclusions

Our data further confirmed the role of ncRNA, especially miRNAs, in the pathogenesis of gliomas, suggesting their potential as both biomarkers and therapeutic targets. Our findings support the hypothesis that certain miRNAs act as key regulators of critical pathways involved in glioma progression, including cell proliferation, apoptosis, invasion, and angiogenesis. Therefore, a better understanding of miRNA-mediated networks may open new avenues for precision medicine in glioma treatment.

## Figures and Tables

**Table 1:** Demographic and clinical characteristics of patients diagnosed with glioma.

Variable	Number of Case (%)
**Gender**	
Male	43 (62.3)
Female	26 (37.7)
**Age (Year)**	
<40 years	3 (4.3)
40–49 years	4 (5.8)
50–59 years	11 (15.9)
60–69 years	34 (49.3)
≥70 years	17 (24.7)
**Symptoms**	
Convulsion	23 (33.3)
Nausea/vomiting	36 (52.2)
Headache	58 (84.1)
Motor deficits	20 (29.0)
Sensory deficits	12 (17.4)
**Medical condition**	
Diabetes	14 (20.3)
Smoking	30 (43.5)
Hypertension	27 (39.1)
**Grade**	
LGGs	17 (24.6)
HGGs	52 (75.4)
**BMI = kg/m^2^**	
Healthy	11 (15.9)
Over-weight	42 (60.9)
Obesity	16 (23.2)
Median KPS (range)	75 (55–100)
Follow-up (range)	33 (12–70)

**Table 2:** Upregulated and downregulated miRNAs in HGG versus NAT.

Upregulated miRNAs	miRBase Accession	Genomic Location	Log2FC	FDR	*p*-Value
hsa-miR-1306-5p	MIMAT0022726	22q11.21	2.123	0.0629	0.00021
hsa-miR-21	MIMAT0000076	17q23.1	1.242	0.0100	0.00102
hsa-miR-1321	MIMAT0005952	Xq21.2	1.121	0.7421	0.00172
hsa-miR-1275	MIMAT0005929	6p21.31	2.574	0.0392	0.00578
hsa-miR-221	MIMAT0000278	Xp11.3	1.432	0.0072	0.00635
hsa-miR-374a-5p	MIMAT0000727	Xq13.2	1.845	0.0527	0.00891
hsa-miR-1301-5p	MIMAT0026639	2p23.3	3.321	0.0017	0.03281
hsa-miR-10b-5p	MIMAT0000254	2q31.1	1.734	0.0063	0.03762
hsa-miR-1270	MIMAT0005924	19p12	2.543	0.0261	0.04852
hsa-miR-130b-5p	MIMAT0004680	22q11.21	1.536	0.0264	0.05000
hsa-miR-130a-5p	MIMAT0004593	11q12.1	1.492	0.0782	0.05021
**Downregulated miRNAs**	**miRBase accession**	**Genomic location**	**Log2FC**	**FDR**	***p*-value**
hsa-miR-328-5p	MIMAT0026486	16q22.1	−3.429	0.1107	0.00004
hsa-miR-210	MI0000286	11p15.5	−1.534	0.0152	0.00009
hsa-miR-222-3p	MIMAT0000279	Xp11.3	−4.374	0.01	0.00021
hsa-miR-1	MI0000651	20q13.33	−1.523	0.01	0.00025
hsa-miR-1271-5p	MIMAT0005796	5q35.2	−2.536	0.0207	0.00025
hsa-miR-181a-5p	MIMAT0000256	9q33.3	−2.932	0.0635	0.00034
hsa-miR-1323	MIMAT0005795	19q13.42	−3.023	0.0523	0.00042
hsa-miR-133a	MI0000450	18q11.2	−2.936	0.01	0.00043
hsa-miR-103a-5p	MIMAT0009196	20p13	−2.736	0.0972	0.00082
hsa-miR-128a	MI0000447	2q21.3	−1.563	0.4521	0.00312
hsa-miR-124-3p	MIMAT0000422	8p23.1	−4.182	0.0746	0.00345
hsa-miR-487a	MI0002471	14q32.31	−1.837	0.032	0.00362
hsa-miR-218	MIMAT0000275	4p15.31	−1.932	0.01	0.00386
hsa-miR-365b-5p	MIMAT0022833	17q11.2	−2.832	0.01	0.00394
hsa-miR-220a	MI0000297	Xq25	−4.832	0.0072	0.00439
hsa-miR-516b-2	MI0003167	19q13.42	−2.051	0.0829	0.00482
hsa-miR-154	MI0000480	14q32.31	−2.539	0.0142	0.00536
hsa-miR-138-5p	MIMAT0000430	11q13.1	−2.728	0.0293	0.00632
hsa-miR-29a	MI0000087	7q32.3	−1.643	0.01	0.00635
hsa-miR-212-3p	MIMAT0000269	17p13.3	−3.682	0.01	0.00693
hsa-miR-92a-1	MI0000093	13q31.3	−2.543	0.0791	0.00735
hsa-miR-361-5p	MIMAT0000703	Xq21.2	−1.721	0.01	0.00792
hsa-miR-128-5p	MIMAT0026477	2q21.3	−3.932	0.01279	0.00835
hsa-miR-185-5p	MIMAT0000455	22q11.21	−1.231	0.1632	0.00873
hsa-miR-423-5p	MIMAT0004748	17q11.2	−2.239	0.0425	0.00935
hsa-let-7c-5p	MIMAT0000064	21q21.1	−1.374	0.01	0.01118
hsa-miR-27b	MI0000440	9q22.32	−1.592	0.01	0.01232
hsa-miR-181a-2-3p	MIMAT0004558	9q33.3	−4.392	0.01	0.03401
hsa-miR-184	MIMAT0000454	15q25.1	−2.431	0.0195	0.03628
hsa-miR-99a	MI0000101	21q21.1	−3.231	0.0112	0.04362
hsa-miR-376a-1	MI0000784	14q32.31	−1.119	0.0526	0.04382
hsa-miR-139-5p	MIMAT0000250	11q13.4	−1.293	0.01	0.04852
hsa-miR-379-5p	MIMAT0000733	14q32.31	−1.572	0.01	0.05001

**Table 3:** Predicted target genes of the selected miRNAs.

miRNAs	Target Gene (Symbol)	Accession Number	Genomic Location	Description
miR-21	*ZNF367*	ENSG00000165244	9q22.32	Zinc finger protein 367
	*NFAT5*	ENSG00000102908	16q22.1	Nuclear factor of activated T cells 5
	*PIK3R1*	ENSG00000145675	5q13.1	Phosphoinositide-3-kinase regulatory subunit 1
miR-221	*CDK6*	ENSG00000105810	7q21.2	Cyclin-dependent kinase 6
	*TP53I11*	ENSG00000175274	11p11.2	Tumor protein p53 inducible protein 11
	*VPS53*	ENSG00000141252	17p13.3	GARP complex subunit
miR-1306-5p	*TET3*	ENSG00000187605	2p13.1	Tet methylcytosine dioxygenase 3
	*NEPRO*	ENSG00000163608	3q13.2	Nucleolus and neural progenitor protein
	*NPTXR*	ENSG00000221890	22q13.1	Neuronal pentraxin receptor
miR-1321	*KLK4*	ENSG00000167749	19q13.3	kallikrein-related peptidase 4
	*NQO1*	ENSG00000181019	16q22.1	NAD(P)H quinone dehydrogenase 1
	*SERPINA1*	ENSG00000197249	14q32.13	Serpin peptidase inhibitors
miR-374a-5p	*CADM2*	ENSG00000175161	3p12.1	Cell adhesion molecule 2
	*NLN*	ENSG00000123213	5q12.3	Neurolysin
	*ZNF519*	ENSG00000175322	18p11.21	Zinc finger protein 519

## Data Availability

The original contributions presented in this study are included in the article. Further inquiries can be directed to the corresponding authors.

## Abbreviations

The following abbreviations have been used in this manuscript:

cDNA	Complementary DNA
CNS	Central Nervous System
FC	Fold Change
FDR	False Discovery Rate
FFPE	Formalin-Fixed Paraffin-Embedded
GMB	Glioblastoma Multiforme
HGG	High-grade Glioma
LGG	Low-grade Glioma
lncRNA	Long-Non-Coding RNA
miRNA	MicroRNA
NAT	Normal Adjacent Tissue
ncRNA	Non-Coding RNA
NGS	Next Generation Sequencing
PCA	Principal Component Analysis
piRNA	Piwi RNA
PRC	Polycomb Repressive Complex
RNP	Ribonucleoprotein
RNAP	RNA Polymerase II
RPM	Reads Per Million
RT-qPCR	Real-Time Quantitative PCR
rRNA	Ribosomal RNA
siRNA	Small Interfering
snRNA	Small Nuclear RNA
snoRNA	Small Nucleolar RNA
sRNA	Small RNA
TCGA	The Cancer Genome Atlas
tRNA	Transfer RNA
UMI	Unique Molecular Identifier
WHO	World Health Organization
